# Genetic Structure and Forensic Feature of 38 X-Chromosome InDels in the Henan Han Chinese Population

**DOI:** 10.3389/fgene.2021.805936

**Published:** 2022-01-03

**Authors:** Lin Zhang, Zhendong Zhu, Weian Du, Shengbin Li, Changhui Liu

**Affiliations:** ^1^ Bio-evidence Science Academy, Xi’an Jiaotong University, Xi’an, China; ^2^ Department of Forensic Medicine, Xinxiang Medical University, Xinxiang, China; ^3^ Department of Human Anatomy, School of Basic Medical Sciences, Xinxiang Medical University, Xinxiang, China; ^4^ HOMY GeneTech Incorporation, Foshan, China; ^5^ Guangzhou Forensic Science Institute, Guangzhou, China

**Keywords:** Henan Han population, forensic features, population genetics, InDel, X-chromosome

## Abstract

Insertion/deletion (InDel) polymorphisms, as ideal forensic markers, show useful characteristics of both SNPs and STRs, such as low mutation rate, short amplicon size and general applicability of genotyping platform, and have been used in human identification, population genetics and biogeographic research in recent years. X-chromosome genetic markers are significant in population genetic studies and indispensable complements in some complex forensic cases. However, the population genetic studies of X-chromosome InDel polymorphisms (X-InDels) still need to be explored. In this study, the forensic utility of a novel panel including 38 X-InDel markers was evaluated in a sample of Han population from Henan province in China. It is observed that the heterozygosities ranged from 0.0054 to 0.6133, and the combined discrimination power was 1–9.18 × 10^−17^ for males and 1–7.22 × 10^−12^ for females respectively. The mean exclusion chance in trios and duos were 0.999999319 and 0.999802969 respectively. Multiple biostatistics methods, such as principal component analysis, genetic distances analysis, phylogenetic reconstruction, and structure analysis was used to reveal the genetic relationships among the studied Henan Han group and other 26 reference groups from 1,000 Genomes Project. As expected, the Henan Han population was clustered with East Asian populations, and the most intimate genetic relationships existed in three Han Chinese populations from Henan, Beijing and South China, and showed significant differences compared with other continental groups. These results confirmed the suitability of the 38 X-InDel markers both in individual identification and parentage testing in Han Chinese population, and simultaneously showed the potential application in population genetics.

## Introduction

Forensic genetic research is devoted to the pursuit of ideal genetic markers that can be used to the routine forensic casework including individual identification, paternity and kinship testing, race and species identification, biological ancestry analysis et al. Short tandem repeat markers (STRs) have been widely used in routine forensic casework. However, due to the defects of high spontaneous mutation rate, the limitation of detecting degraded samples, stutter peak et al., STRs occasionally produced inconclusive results, especially in some deficient paternity cases or some cases with only degraded tissue as DNA source (Huang et al., 2002; Fan and Chu, 2007; Giardina et al., 2011; Caputo et al., 2017). To overcome the abovementioned defects of STRs, new genetic markers have been searching by scholars worldwide in recent years. As a kind of special dimorphic marker, Insertion/Deletion polymorphisms (InDels) are generated in natural populations by inserting or deleting DNA fragments of different sizes and widely distributed throughout the genome (Weber et al., 2002; Mills et al., 2006). Although InDel markers have fewer genetic diversity than STRs, they show lower mutation rates and are more suitable for detecting degraded DNA samples because analysis can be based on short amplicons which increases the chance to generate more accurate data with high resolution (Gomes C. et al., 2020). Furthermore, InDel polymorphisms are length polymorphism markers which can be easily separated in capillary electrophoresis technology. Hence, InDels are considered to be ideal forensic markers and have attracted attentions from forensic researchers worldwide (Sheng et al., 2018). InDels have been increasingly explored and used in forensic genetics and biogeographical ancestry inferences, and much more extensive forensic and population genetic studies are ongoing (Bastos-Rodrigues et al., 2006; Larue et al., 2014; Du et al., 2019; He et al., 2019).

X-chromosome markers recombine along the whole chromosome during female meiosis in females and are transmitted to both female and male descendants, however, are entirely transmitted to female offspring in males (Gomes I. et al., 2020). Due to their distinctive transmission properties, X-chromosome markers have emerged as a powerful complementary tool of parentage testing, especially in some special parentage cases such as “half-siblings”, “avuncular” or “grandparent-grandchild” (Gomes et al., 2012). In addition, for the smaller effective population size, the X-chromosome shows faster genetic drift than autosomes, hence genetic distances between populations are significantly larger on the X chromosome (Schaffner, 2004). These specific properties of X-chromosome makes it a good system for assessing admixture of population and investigating evolutionary anthropology.

Han Chinese who makes up 91.50% of Chinese population has shown the regional genetic diversities because of national amalgamation in history (Lin et al., 2017; Cao et al., 2020). Han Chinese originated from Huaxia tribes or the Neolithic Yan Huang tribes along the upper and middle Yellow River in northern China (Wen et al., 2004). As the center birthplace of Huaxia civilization, Henan province, with a total population of 99.37 million, of which 98.84% are Han Chinese, enjoys the representative group of Han Chinese in China. In this study, a novel multiplex amplification system including 38 X-InDel loci was tested in Henan Han Chinese population. We firstly genotyped 38 X-InDel loci in 268 individuals of Han Chinese from Henan province and calculated the forensic parameters to provide basic group data for paternity identification and individual identification. Moreover, using the 38 X-InDel loci, we analyzed the population genetic differentiations between Henan Han population and other 26 reference populations from 1,000 Genomes Project.

## Materials and Methods

### Sample Collection

Our study was approved by the Ethics Committee of Xi’an Jiaotong University Health Science Center (No.2021-1,444). Each volunteer signed an informed consent, giving his permission to the analysis and publishing of anonymized genetic data obtained from his biological sample. A total of 268 fingertip blood samples were gathered from unrelated individuals (106 females and 162 males) of Chinese Han population in Henan province (HNC). The authentication was performed to ensure those volunteers are indigenous people of Henan province. All the volunteers meet the conditions that the past three generations are Han group and have no trans-regional migration.

**FIGURE 1:**
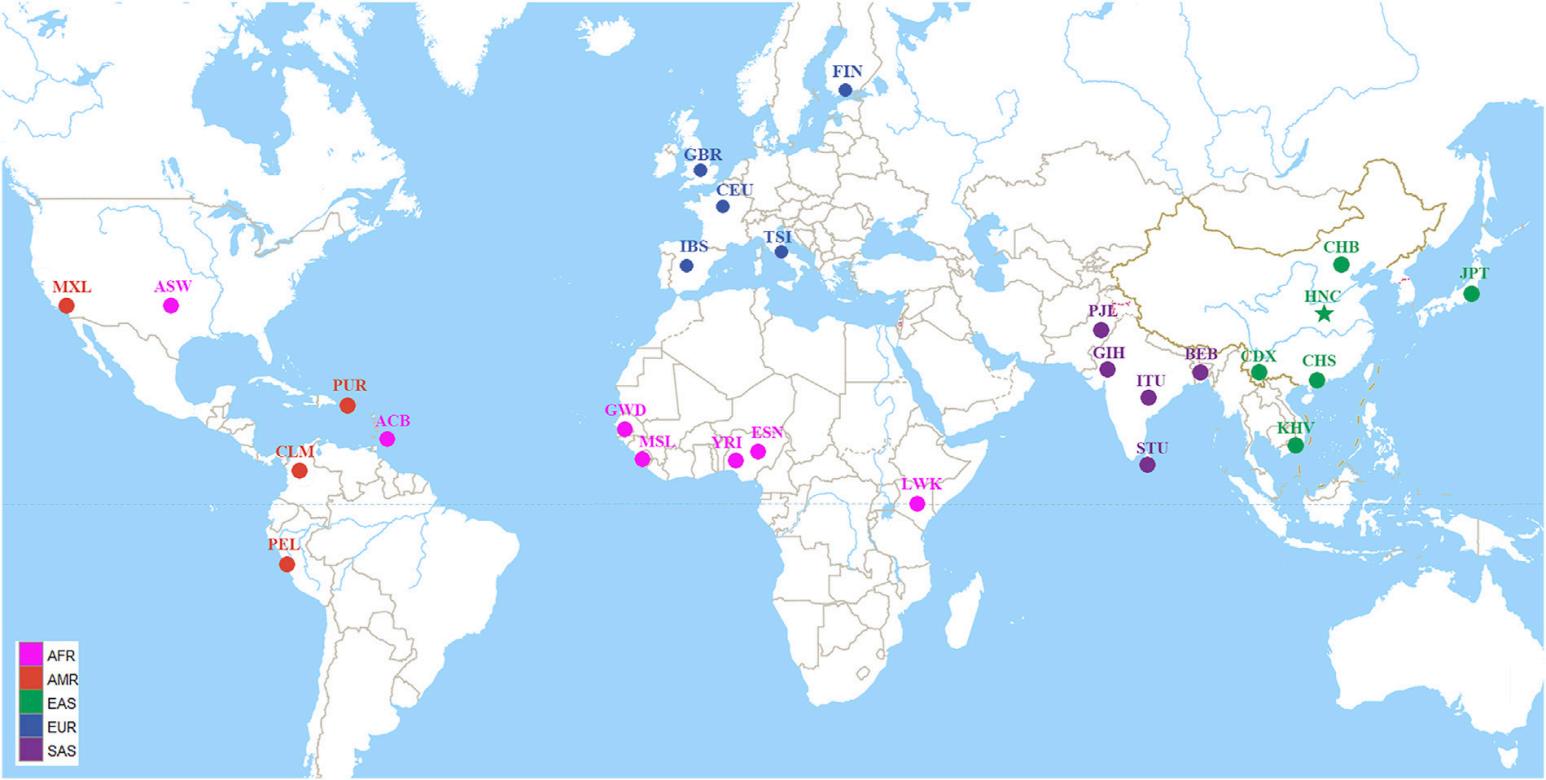
Geographic location information of the studied population and other 26 worldwide reference populations. The 27 populations were divided into five continental groups: African (AFR) including African ancestry in Southwest United States (ASW), African Caribbean in Barbados (ACB), Esan in Nigeria (ESN), Gambian in Western Division-Mandinka (GWD), Luhya in Webuye, Kenya (LWK), Mende in Sierra Leone (MSL), Yoruba in Ibadan, Nigeria (YRI); American (AMR) including Colombian in Medellín, Colombia (CLM), Mexican Ancestry in Los Angeles CA United States (MXL), Peruvian in Lima, Peru (PEL), Puerto Rican in Puerto Rico (PUR); East Asian (EAS) including Chinese Dai in Xishuangbanna (CDX), Han Chinese in Beijing, China (CHB), Han Chinese in South China (CHS), Henan Han Chinese (HNC), Japanese in Tokyo, Japan (JPT), Kinh in Ho Chi Minh City, Vietnam (KHV); European (EUR) including Utah residents with Northern and Western European ancestry (CEU), Finnish in Finland (FIN), British from England and Scotland (GBR), Iberian populations in Spain (IBS), Toscani in Italia (TSI); South Asian (SAS) including Bengali in Bangladesh (BEB), Gujarati Indians in Houston, Texas, United States (GIH), Indian Telugu in the United Kingdom (ITU), Punjabi in Lahore, Pakistan (PJL), Sri Lankan Tamil in the United Kingdom (STU).

The blood samples were placed in the sample acquisition card and then were dried and stored at room temperature. Meanwhile, we collected 38 InDels raw data and allele frequencies of 26 populations worldwide spanning five continents from the 1,000 Genomes Project (Ensembl Genome Browser, http://grch37.ensembl.org/Homo_sapiens/Info/Index), as shown in Figure 1 (The 1000 Genomes Project Consortium et al., 2010).

### PCR Amplification

All blood samples were amplified directly without DNA extraction. The 268 unrelated samples were genotyped using our new established multiplex amplification system on the Thermo 96-Well PCR System (Thermo Fisher Scientific Company, Carlsbad, United States). The analyzed panel including 38 X-InDel markers, namely rs10671504, rs11277082, rs1160845, rs143123845, rs149102585, rs16367, rs16368, rs16397, rs16637, rs17394, rs199731653, rs2307707, rs2307741, rs2308033, rs2308280, rs25581, rs3048996, rs3077884, rs3215490, rs34763847, rs35574346, rs35954471, rs363794, rs4030406, rs45449991, rs56820033, rs57608175, rs57843641, rs58595330, rs59605609, rs60283667, rs71671860, rs3216913, rs10699224, rs3859989, rs61260787, rs36094418 and rs79829945. The localizations of the different markers were previously described in our published article (Chen et al., 2021). Separation of PCR-amplified products were performed on the ABI 3130xL DNA Analyzer (Applied Biosystems, Foster City, CA, United States). Electropherogram analysis and allele assignment were performed with GeneMapper v 4.0.

### Statistical Analysis

Arlequin v3.5 software was used to test for the linkage disequilibrium (LD) and Hardy-Weinberg Equilibrium (HWE) between all pairs of the 38 X-InDel loci, and *p* values were corrected by the Bonferroni procedure (Excoffier et al., 2007). HWE was evaluated in females, whereas LD was tested by an extension of Fisher’s exact test on contingency tables, D′ and Chi-square values from male haplotype counts (Caputo et al., 2017). LD was considered positive when D’≥0.8 and at distances of ≤60 kb (Reich et al., 2001). Allele frequencies and forensic parameters such as discrimination power (DP), probability of exclusion of trios-testing (PEtrio), probability of exclusion of duos-testing (PEduos), polymorphic information content (PIC) and expected heterozygosity (He) of the 38 X-InDel loci were calculated using StatsX v2.0 software (Lang et al., 2019). Fisher’s exact test were used by SPSS 25.0 software package for comparing allele frequencies between males and females. A value of *p* < 0.05 was considered statistically significant. Chi-square tests were used to compare the allele frequencies between reference populations and Henan Han population.

Unbiased Nei’s genetic distances were calculated in Genalex v6.5 (Peakall and Smouse, 2012) based on allele frequencies of the 38 X-InDel loci and heatmaps were plotted using Pheatmap package of R Statistical Software v4.0.2. The principal component analyses (PCA) were performed using the online tool (https://www.omicstudio.cn/tool). Phylogenetic neighbor-joining (NJ) tree was reconstructed by MEGA software v7.0 using Nei genetic distance matrices. On the basis of raw data of female individuals, population genetic structure was analyzed by the Structure version 2.3.4.21 (Evanno et al., 2005). Structure parameter was set to run 15 replicates from K = 2 to K = 8 with 10,000 burn-ins and 10,000 Markov Chain Monte Carlo (MCMC). CLUMPP version 1.1.2 was used to align the different replicates of STRUCTURE analysis (Jakobsson and Rosenberg, 2007). DISTRUCT 1.1 was used for the graphical display of population structure (Rosenberg, 2004).

## Results and Discussion

### Allele Frequencies

**FIGURE 2:**
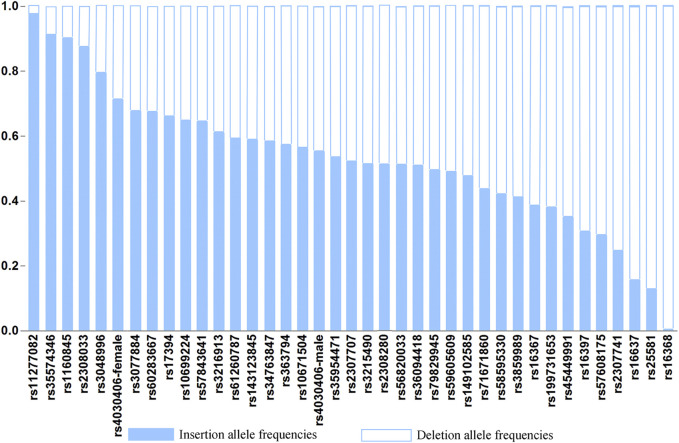
Histogram of allele frequencies at the 38 X-InDel loci of the Henan Han population.

The raw genotype data of 38 X-InDel loci for 268 individuals of Henan Han population are shown in Supplementary Table S1. The insertion allele frequencies of the 38 X-InDel loci are shown in Figure 2. The numerical values of insertion and deletion allele frequencies are shown in Supplementary Table S2.

To evaluate the differences in allele frequencies of the X-InDel loci between males and females, we first carried out fisher’s exact test, and found that there were no statistical significance between males and females (*p* > 0.05) except for rs4030406 locus (*p* = 0.007). The male and female allele frequencies of rs4030406 locus are shown respectively, and the male and female allele frequencies of the other 37 X-InDel loci were combined for calculation. Unless otherwise specified, the female allele frequency of rs4030406 locus is used for calculation. The 29 insertion allele frequencies out of 38 loci for Henan Han population range from 0.3000 to 0.7000. As a whole, the allele frequency distributions of the 38 X-InDel loci are relatively balanced in Henan Han population.

### Linkage Disequilibrium Analysis of 38 X-InDels

**FIGURE 3:**
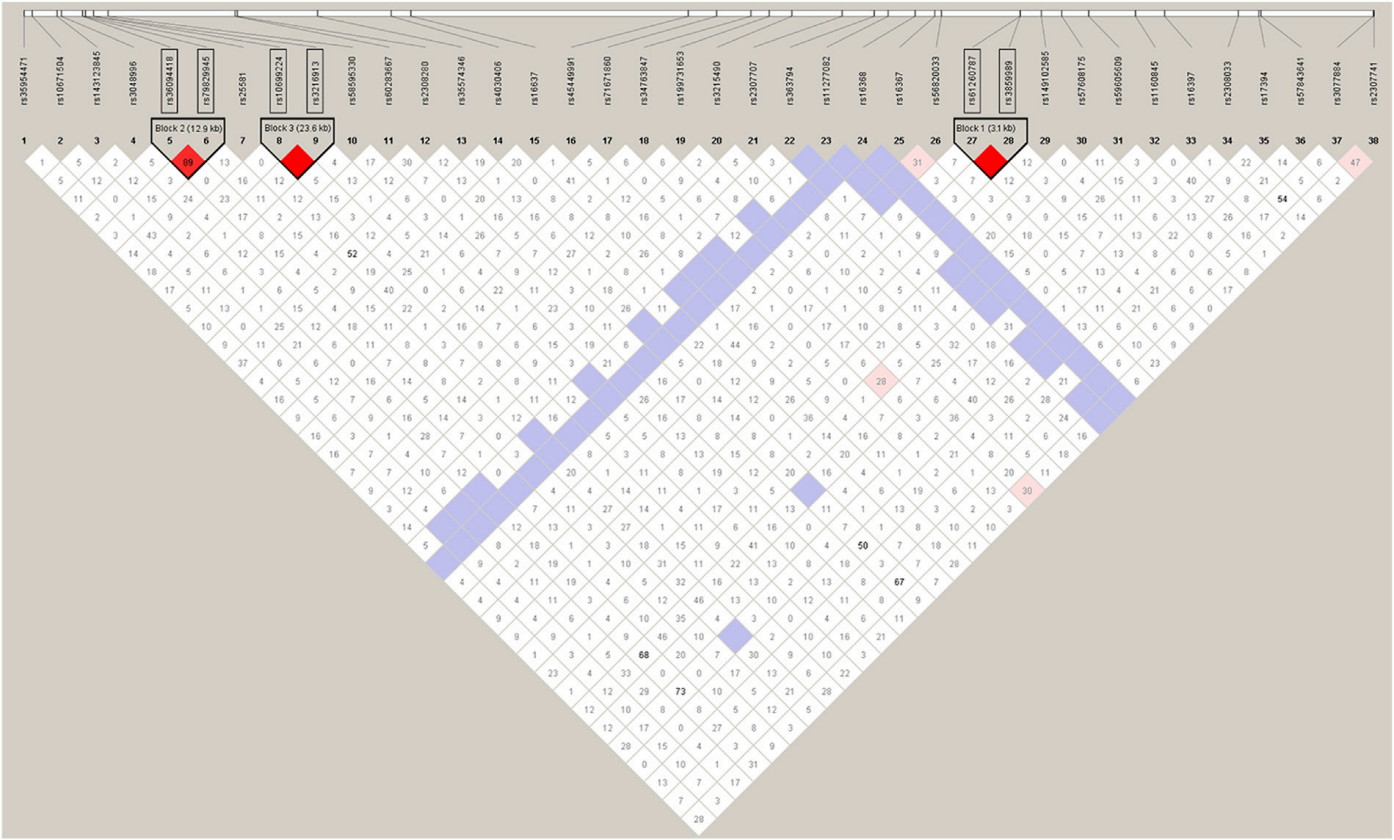
The degree of LD among the 38 InDel loci in Henan Han population. The red color indicates a high level of linkage between two loci.

The recombination rate for the X chromosome is almost exactly two-thirds of the genome average (Kong et al., 2002). It is expected that linkage disequilibrium (LD) will be greater on the X chromosome, especially among younger loci, which have had less time for recombination to break down LD. Therefore, we carried out LD tests firstly and illustrated the degree of LD between the polymorphic loci, as shown in Figure 3. With the condition of D’≥0.8, the linkage Chi-square test was significant (after Bonferroni adjustments) and the distance between the loci is less than 60kb, three disequilibrium linkage blocks containing two linked loci were found in the tests, namely block 1 (rs3859989 and rs61260787 3.1 kb apart), block 2 (rs36094418 and rs79829945 12.9 kb apart), block 3 (rs3216913 and rs10699224 23.6 kb apart). The two loci in each block were combined for the subsequent forensic studies. Meanwhile, we performed LD tests within continental groups (Supplementary Figure S1), and achieved consistent results in East Asian, European and American groups. In South Asian and African groups, the block 1 (rs3859989 - rs61260787) and block 3 (rs3216913 - rs10699224) were detected as well, but no significant association was observed between InDels rs36094418 and rs79829945 (block 2). This discrepancy could be explained by other factors than linkage, such as admixture and population substructure (Martinez et al., 2019). Drawing on the methods described by Ferragut et al. (2017) we retained all the associated loci in the subsequent genetic analysis.

### Forensic Parameters

**FIGURE 4:**
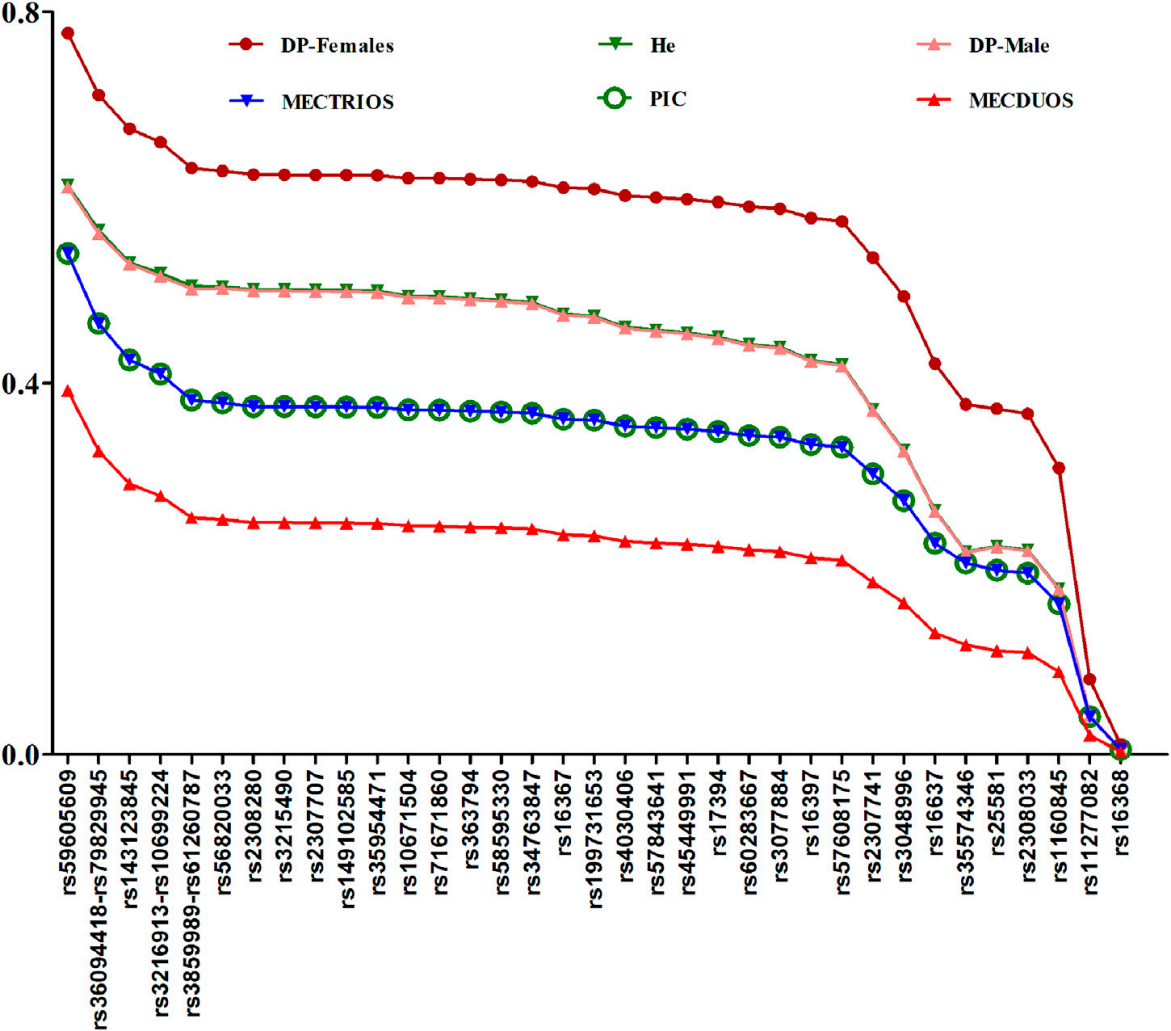
Plots of forensic parameters at the 38 InDel loci in Henan Han population.

To ensure samples can represent the population, Hardy-Weinberg equilibrium (HWE) testing was performed, and no deviations from HWE (*p* > 0.05) were found among the 38 X-InDel loci, which indicated that we can estimate both the forensic characteristics of the single locus (except for the three blocks) and the combined forensic efficiency of the 38 X-InDel loci in our following analysis. Forensic parameters for the 38 X-InDel loci in Henan Han population are shown in Figure 4 and Supplementary Table S3. Most of He values for Henan Han population are in the range of 0.4249–0.6133 (29 out of 35), and most of PIC values for Henan Han population are in the range of 0.3–0.5399 (29 out of 35). The combined DP (CDP) for males and females are 1–9.18E-17 and 1-7.22E-12 respectively. The mean exclusion chance in trios and duos are 0.999999319 and 0.999802969, respectively. Based on the standard of 0.9999, the results indicated that the panel of 38 X-InDel loci meets the efficiency of individual identification and parentage testing of trios in Henan Han population. It is a pity that the relative standard deviations of the mean exclusion chance in duos is 0.0001 lower. We speculate that replacing the low heterozygous loci and linked loci or adding more high heterozygous loci may achieve the standard of 0.9999. On the whole, the panel of 38 X-InDel loci can be used as an effective supplementary tool for human identity and parentage testing in China.

### Allele Frequency Distributions in 27 Populations

**FIGURE 5:**
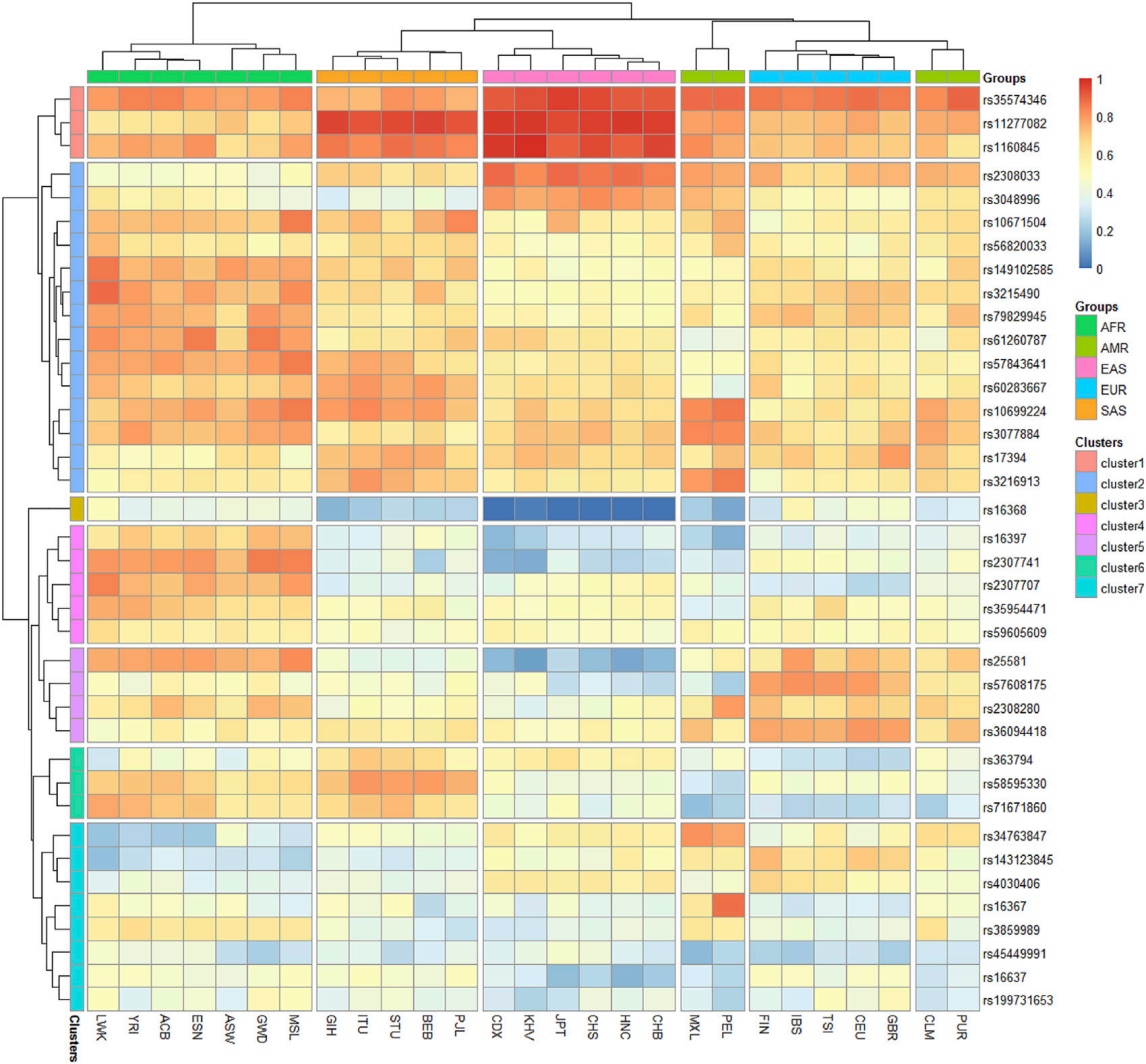
Heatmap on the basis of the insertion allele frequency distributions for Henan Han group and other 26 populations worldwide.

To investigate the allele frequency distributions of the 38 X-InDel loci in different continental populations, we further collected the raw data of the 38 X-InDel loci in 26 populations worldwide and conducted a heatmap based on the insertion allele frequencies of the 38 X-InDel loci of the 26 populations and Henan Han population, as shown in Figure 5. Red represented high insertion allele frequencies, on the contrary, blue represented low insertion allele frequencies. The populations from Africa, America, East Asia, Europe and South Asia were divided into five groups roughly according to the continent, except for the Puerto Rican (PUR) and Colombian (CLM) which were clustered with the European populations, and Henan Han population was classified to East Asian population. After clusters analyzing, we divided the 38 X-InDel loci into seven clusters (1–7). Cluster 1, including rs35574346, rs11277082 and rs1160845 loci, shows high allele frequencies in East and South Asian population (>0.9). Instead, the cluster three that contains only one locus of rs16368 is observed at low insertion allele frequencies in five groups especially in East Asian population (<0.1). Cluster two contains 14 loci, which have high heterozygosity and polymorphism in the 27 populations and most of the insertion allele frequencies of the 14 X-InDel loci are between 0.4–0.6. The insertion allele frequencies of clusters 4–7 show more differences among groups. Markers showing large allele frequency differences between different ancestral or geographically distant populations were considered to be ideal ancestry informative markers (AIMs). We found rs11277082 and rs1160845 loci in Asian population, rs2307741 locus in African population, rs16367 locus in Peruvian and rs34763847 locus in Peruvian and Mexican showed high frequencies. In contrast, rs16368 locus and rs25581 locus showed low frequencies in East Asian population. These results mean that the seven loci could be eminently suitable for AIMs.

Overall, the vast majority of loci in the panel including 38 X-InDel loci shows better heterozygosities and polymorphisms, especially in African, American and European populations, and the difference among groups are valuable in understanding the ethnic aggregation and migration and forensic identifications.

### Genetic Affinity Analysis Among the 27 Populations Using the Panel of 38 X-InDel Loci

**FIGURE 6:**
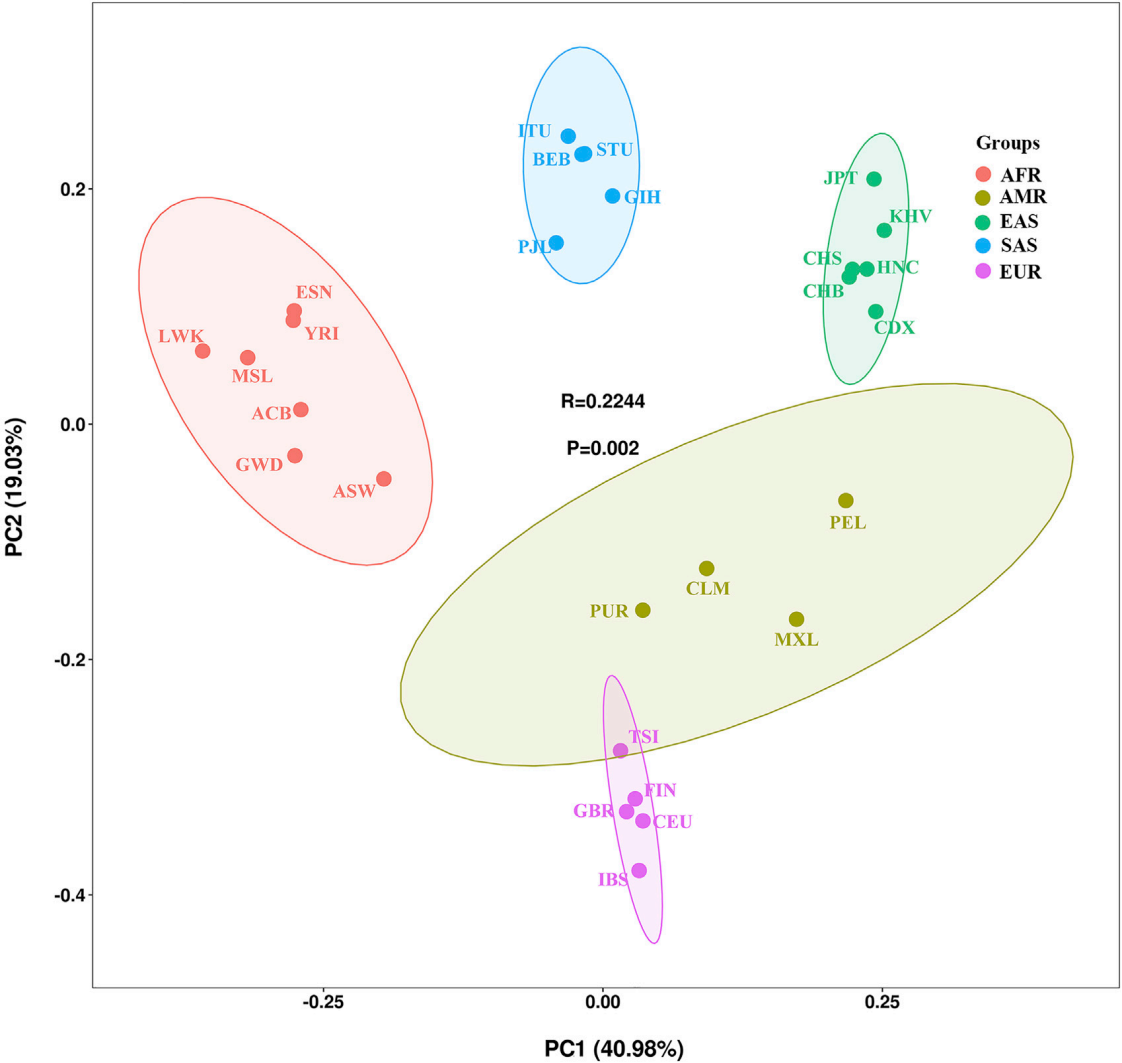
PCA among the 27 populations based on the allele frequencies of 38 X-InDel markers.

To illustrate the genetic background and population relationship of the 27 populations using the 38 X-InDel markers, the PCA was performed based on insertion allele frequencies. As shown in Figure 6, the 27 populations were labeled with five different colors according to the large continental-level groups, and the contribution rates of PC1 and PC2 were 40.98 and 19.03% respectively, which explained aggregately about 60% of the genetic structure variances among populations. Consistent with the large continental-level groups, we observed the five main population clusters. We noticed that the American populations clustered not so well as other four groups and showed genetic affinities to the European populations. Li et al. also showed that Colombian was clustered with European populations and other American populations were dispersedly distributed in MDS plot (Li et al., 2019). For historical reasons, most of the Puerto Rican and Colombian from the four American populations are European or European-Indian mixed-race population (Via et al., 2011; Ossa et al., 2021), which could be the reason that American populations clustered not obviously.

Although East Asian populations cluster tightly in the upper right corner, they showed substantial substructure in the PC2. Han Chinese (CHB, CHS, HNC), Japanese, Chinese Dai and Vietnamese Kinh were separated each other. The studied Henan Han population was plotted in the middle of the East Asian populations and presented close genetic affinities to Han Chinese in Beijing and Han Chinese in South China.

**FIGURE 7:**
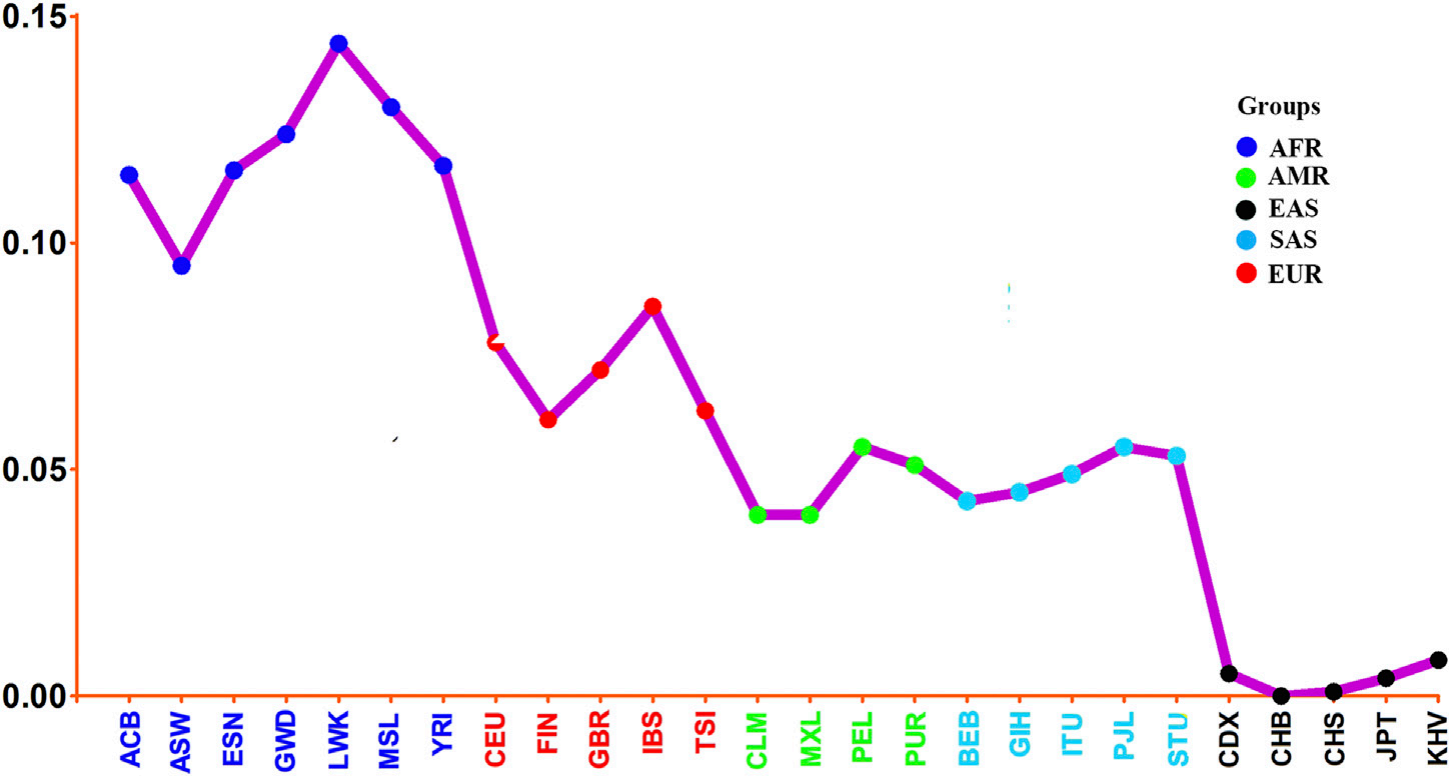
Line chart on the basis of Nei’s genetic distances between studied Henan Han population and the other 26 reference populations.

To further reveal population genetic similarities and divergences of Henan Han population and other 26 reference populations, the pairwise Nei’s genetic distances between studied Henan Han population and the other 26 reference populations were calculated and the result was shown in the form of line chart (Figure 7). We observed that Henan Han population had close affinities with East Asian populations (with smallest genetic distances), especially with Han Chinese in Beijing (CHB, 0.000) and Han Chinese in South China (CHS, 0.001), and showed most distant affinities with African populations (with largest genetic distances), especially with Luhya in Webuye, Kenya (with largest genetic distances, 0.144). It should be noted that Henan Han population showed closer affinity with Japanese (JPT, 0.004) than Chinese Dai (CDX, 0.005) and Vietnamese Kinh (KHV, 0.008), and even some research found in PCA that the Japanese individuals are overlapped with Chinese Han populations in North China (Cao et al., 2020). Genetic ancestry is strongly correlated with geography as well as linguistic affiliations (Su et al., 1999; HUGO Pan-Asian SNP Consortium et al., 2009; Watanabe et al., 2019). Chinese Dai in Xishuangbanna is geographically and linguistically close to Southeast Asian. Meanwhile, multiple migration in history affect population composition of East Asian but not Southeast Asian, which maybe the reason why the Japanese showed closer genetic relationship with Henan population in comparison with Chinese Dai and Vietnamese Kinh. Surprisingly, we also noted that American populations and South Asian populations showed similar genetic distances with East Asian, and even more close genetic distances of Colombian (CLM) and Mexican Ancestry in Los Angeles United States (MXL) with East Asian. It happens that there is a similar phenomenon was shown in a recent study (Cao et al., 2020).

**FIGURE 8:**
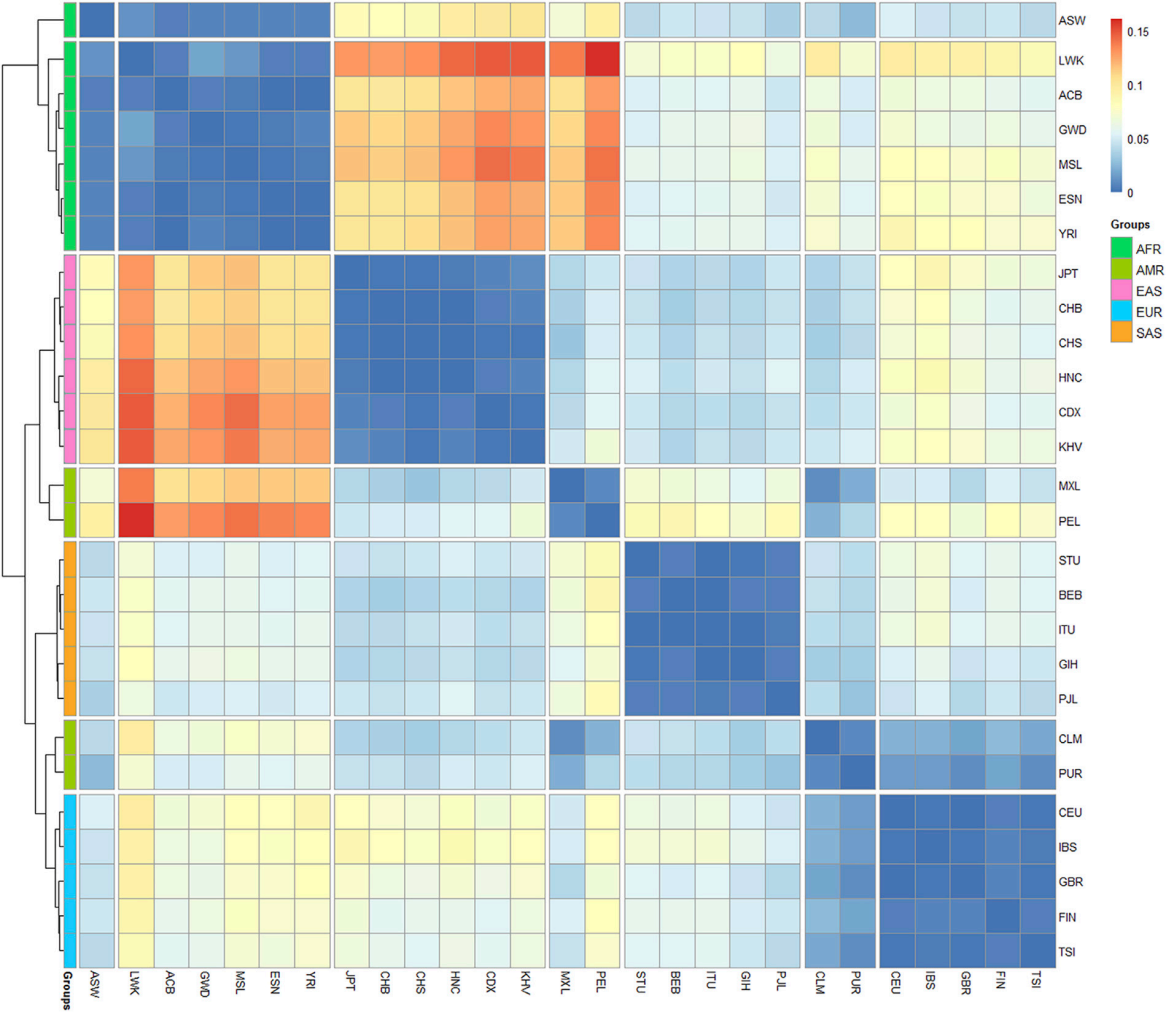
Heatmap on the basis of Nei’s genetic distances among the 27 populations.

Furthermore, the matrix of Nei’s genetic distances was applied to draw a heatmap plot. As shown in Figure 8, red color denotes the larger genetic distances and blue color denotes the smaller genetic distances. We found that the 27 populations were divided into 7 clusters: African populations were split into cluster 1 and 2, and the cluster one contains only one population of African Ancestry in Southwest United States (ASW); American populations were divided into cluster 4 (MXL and PEL) and cluster 6 (CLM and PUR); The clusters 3, five and seven were composed of East Asian populations, South Asian populations and European populations separately. We noted that larger genetic distances were presented between cluster two and clusters 3 and 4, which indicate that most of African populations show distant affinities with East Asian populations and American populations. Meanwhile, we found the smaller genetic distances were presented between cluster six and clusters 4 and 7, indicating that PUR and CLM populations present similar affinities with American populations and European populations.

**FIGURE 9:**
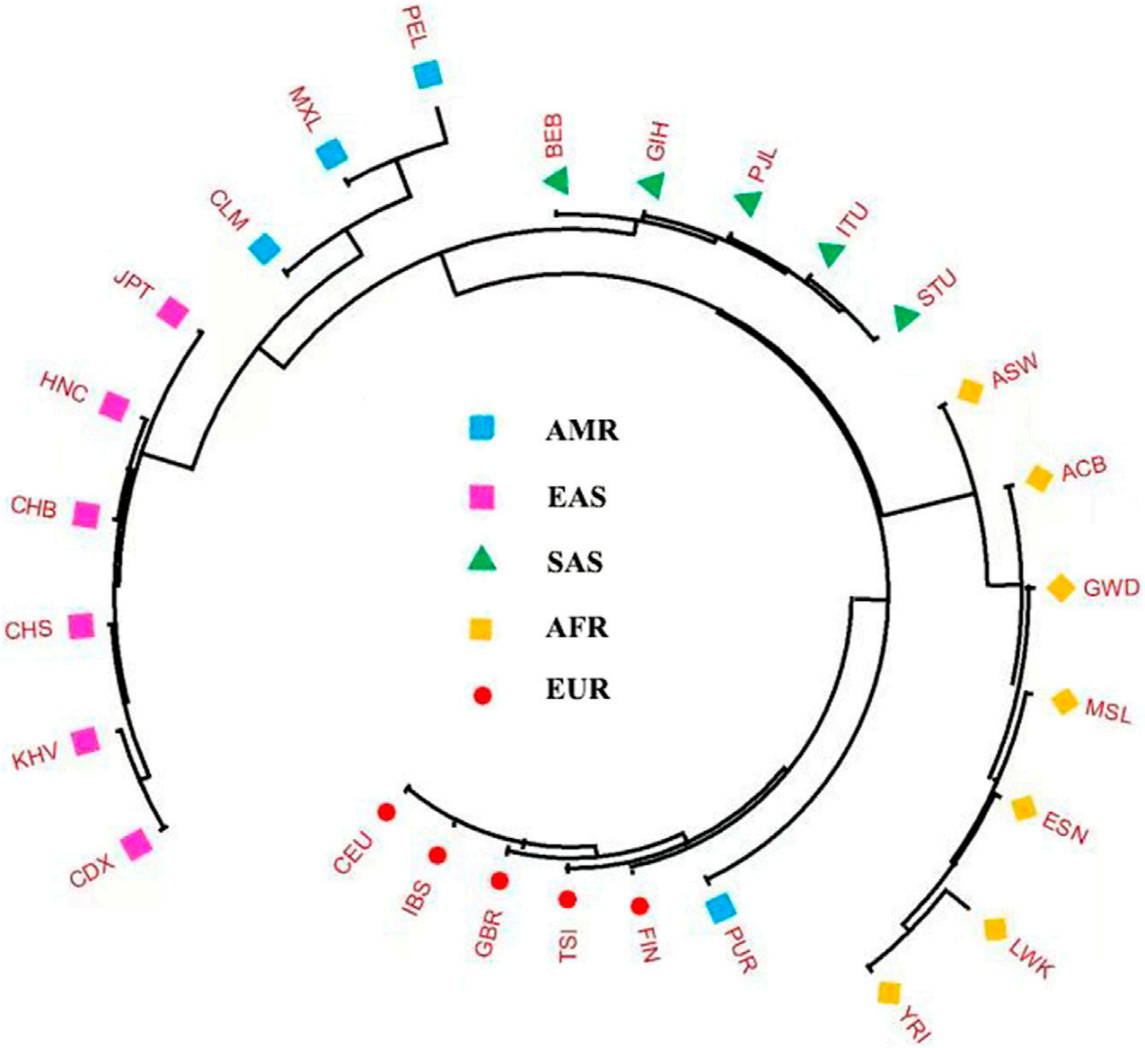
Neighbor-Joining phylogenetic tree constructed on the basis of the Nei’s D matrix among the 27 populations.

To more visually exhibit the genetic similarities and divergences among the 27 populations, a Neighbor-Joining (N-J) phylogenetic tree was constructed based on the allele frequencies of 38 X-InDel loci, as shown in Figure 9. Neighbor-Joining tree supported the population relationship patterns that HNC population was gathered with CHB, and then gathered together with CHS and other East Asian populations. We also found that the Puerto Rican in American populations clustered with the European populations, and the other three American populations showed distant genetic affinities each other. This result supported the aforementioned PCA and Nei’s genetic distances and further proved Puerto Rican have close genetic affinities with the European populations. Similarly, Li et al. showed the close affinities of American populations with European populations and Colombian was clustered with European populations, however, different with our results, the Gujarati Indians in Houston (GIH) was clustered with American populations (Li et al., 2019). In African populations, the population of ASW also showed distant affinities with the other six populations.

**FIGURE 10:**
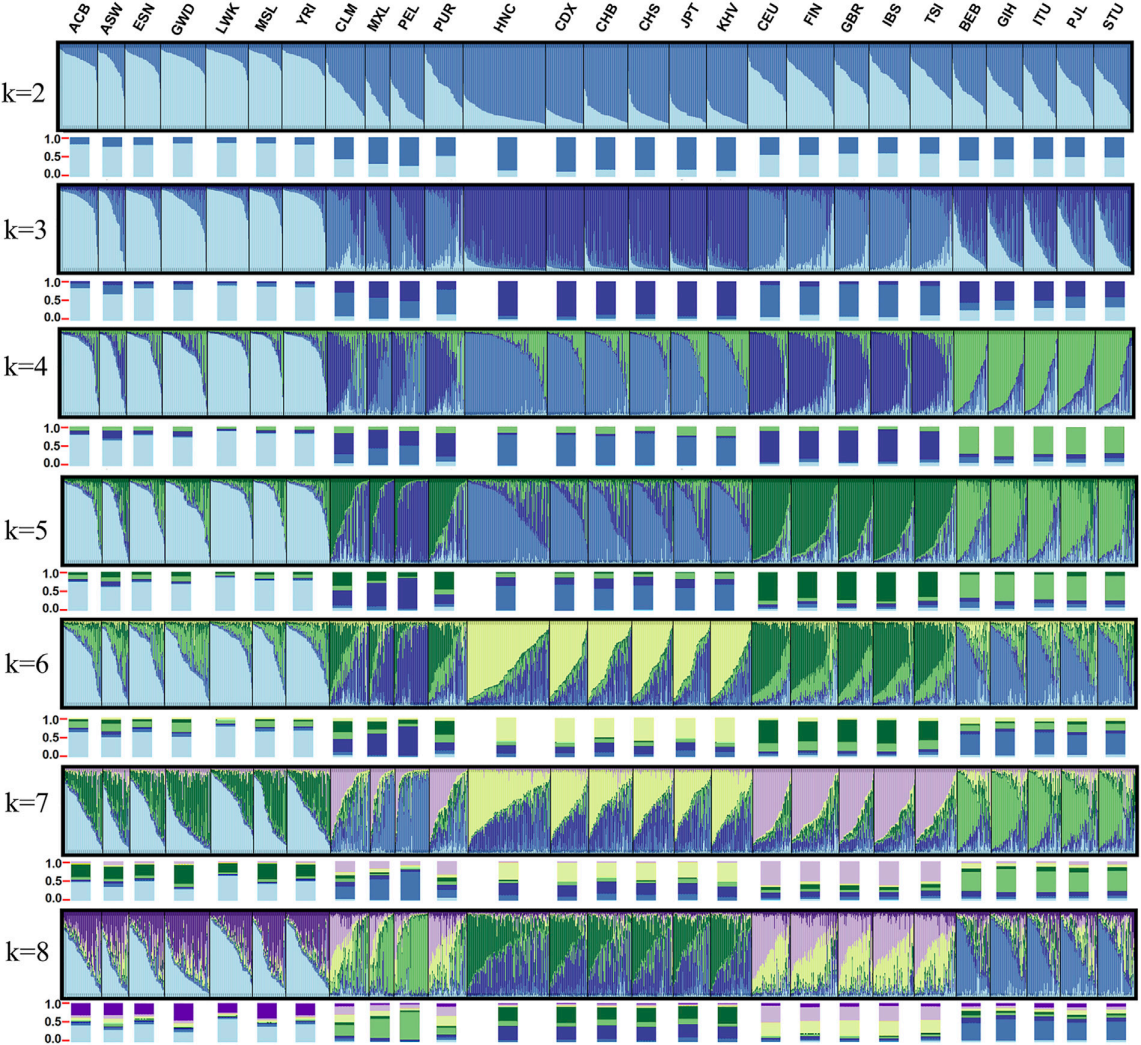
Structure analysis results for females of the 27 populations.

To analyze the population ancestry component among the 27 populations, we conducted structure analysis based on 34 X-InDels (excluding three linked loci and the locus with different allele frequency between males and females) raw genotype of 1,377 females. The detection of the number of genetic groups that best fit the data was performed by an online program STRUCTURE HARVESTER (Earl and Vonholdt, 2012) and the best K-value was observed at K = 4. As shown in Figure 10, K values were set at the range of two–8. Each vertical line represents an individual and all individuals were clustered by the populations. Colors represent the inferred ancestry from K ancestral populations. Histograms below each population show proportions of ancestry components in the population. When K = 2, East Asian-dominant component and African-dominant component were distinguished clearly. When K = 3, European-dominant component was identified, and we could distinguish African, East Asian and South Asian groups based on the proportions of ancestry components, but failure to distinguish European and American group. When K = 4 (the optimum K-value), South Asian-dominant component was further separated clearly. We also observed that the identified four ancestry components were generally in accord with geographic patterns. In addition, when K = 4, Mexican and Peruvian population could be separated with European East Asian, South Asian and African groups. The ancestry components of Colombian and Puerto Rican population were considered to be intermediate between Mexican and Peruvian populations and European populations. When K > 4, no more substructures were found in the 27 populations. The studied Henan Han population shared a similar ancestry component with the other East Asian populations. It has long been recognized that markers with relatively low mutation rates (SNP, InDels) serve as best loci for the analysis of human history over longer time scales (Moriot et al., 2018). However, not as Yuchen Wang et al. described that Han Chinese, Japanese and Korean can be distinguished using a series of single nucleotide polymorphism (SNP) AIMs (Wang et al., 2018), there are no sub-populations were separated in East Asian populations, African populations, European populations and South Asian populations in our study, which could be ascribed to the relative stability of X-Chromosome or more representative loci were needed to distinguish sub-populations within continental populations. We also conducted structure analysis based on raw genotype of 1,395 males and achieved familiar results, as shown in Supplementary Figure S2.

## Conclusion

In the present study, we first described a novel panel of 38 X-InDels and investigated the forensic efficiency in Henan Han population. The results showed that this panel is powerful as a complementary tool for forensic individual identification and parentage testing of trios. Based on the allele frequencies and raw data of the 38 X-InDels, we conducted principal component analysis, calculated Nei’s genetic distances, and constructed phylogenetic tree and structure analysis to illustrate the genetic affinity and population ancestry component among Henan Han population and 26 populations worldwide. It is observed that the grouping from ancestry component analysis and genetic affinity revealed by the panel of 38 X-InDels are generally consistent with geographical classifications. Meanwhile, we noticed that mixed-race populations could be distinguished clearly from the continental population groups by using the panel of 38 X-InDels. However, sub-population identification within continental population groups was failed due to the limitation of X-InDels. It is expected that compound markers which are adopted to infer biogeographic ancestry would improve accurate classification of populations and sub-populations. In conclusion, this study makes a solid foundation of the population data for the application of the 38 X-InDel markers panel in individual identification, parentage testing and biogeographic ancestry analysis, though much more regional and national population data still need to be collected.

## Data Availability

The raw data used during the current study were uploaded as supplementary materials, and all data are fully available without restriction.

## References

[B1] Bastos-Rodrigues L. Pimenta J. R. Pena S. D. J. (2006). The Genetic Structure of Human Populations Studied through Short Insertion-Deletion Polymorphisms. Ann. Hum. Genet. 70, 658–665. 10.1111/j.1469-1809.2006.00287.x 29488221

[B2] Cao Y. Li L. Li L. Xu M. Feng Z. Sun X. (2020). The ChinaMAP Analytics of Deep Whole Genome Sequences in 10,588 Individuals. Cell Res. 30, 717–731. 10.1038/s41422-020-0322-9 32355288 PMC7609296

[B3] Caputo M. Amador M. A. Santos S. Corach D. (2017). Potential Forensic Use of a 33 X-InDel Panel in the Argentinean Population. Int. J. Leg. Med. 131, 107–112. 10.1007/s00414-016-1399-z 27282766

[B4] Chen L. Pan X. Wang Y. Du W. Wu W. Tang Z. (2021). Development and Validation of a Forensic Multiplex System with 38 X-InDel Loci. Front. Genet. 12, 670482. 10.3389/fgene.2021.670482 34484288 PMC8416044

[B5] Du W. Feng C. Yao T. Xiao C. Huang H. Wu W. (2019). Genetic Variation and Forensic Efficiency of 30 Indels for Three Ethnic Groups in Guangxi: Relationships with Other Populations. PeerJ 7, e6861. 10.7717/peerj.6861 31110924 PMC6501771

[B6] Earl D. A. Vonholdt B. M. (2012). STRUCTURE HARVESTER: a Website and Program for Visualizing STRUCTURE Output and Implementing the Evanno Method. Conserv. Genet. Resour. 4, 359–361. 10.1007/s12686-011-9548-7

[B7] Evanno G. Regnaut S. Goudet J. (2005). Detecting the Number of Clusters of Individuals Using the Software STRUCTURE: a Simulation Study. Mol. Ecol. 14, 2611–2620. 10.1111/j.1365-294x.2005.02553.x 15969739

[B8] Excoffier L. Laval G. Schneider S. (2007). Arlequin (Version 3.0): an Integrated Software Package for Population Genetics Data Analysis. Evol. Bioinform Online 1, 47–50. 10.1177/117693430500100003 19325852 PMC2658868

[B9] Fan H. Chu J.-Y. (2007). A Brief Review of Short Tandem Repeat Mutation. Genomics Proteomics Bioinformatics 5, 7–14. 10.1016/s1672-0229(07)60009-6 17572359 PMC5054066

[B10] Ferragut J. F. Bentayebi K. Pereira R. Castro J. A. Amorim A. Ramon C. (2017). Genetic Portrait of Jewish Populations Based on Three Sets of X-Chromosome Markers: Indels, Alu Insertions and STRs. Forensic Sci. Int. Genet. 31, e5. 10.1016/j.fsigen.2017.09.008 28951006

[B11] Giardina E. Spinella A. Novelli G. (2011). Past, Present and Future of Forensic DNA Typing. Nanomedicine 6, 257–270. 10.2217/nnm.10.160 21385128

[B12] Gomes C. Magalhães M. Alves C. Amorim A. Pinto N. Gusmão L. (2012). Comparative Evaluation of Alternative Batteries of Genetic Markers to Complement Autosomal STRs in Kinship Investigations: Autosomal Indels vs. X-Chromosome STRs. Int. J. Leg. Med 126, 917–921. 10.1007/s00414-012-0768-5 22940765

[B13] Gomes C. Quintero-Brito J. D. Martínez-Gómez J. Pereira R. Baeza-Richer C. Aler Gay M. (2020a). Spanish Allele and Haplotype Database for 32 X-Chromosome Insertion-Deletion Polymorphisms. Forensic Sci. Int. Genet. 46, 102262. 10.1016/j.fsigen.2020.102262 32088644

[B14] Gomes I. Pinto N. Antão-Sousa S. Gomes V. Gusmão L. Amorim A. (2020b). Twenty Years Later: A Comprehensive Review of the X Chromosome Use in Forensic Genetics. Front. Genet. 11, 926. 10.3389/fgene.2020.00926 33093840 PMC7527635

[B15] He G. Ren Z. Guo J. Zhang F. Zou X. Zhang H. (2019). Population Genetics, Diversity and Forensic Characteristics of Tai-Kadai-Speaking Bouyei Revealed by Insertion/deletions Markers. Mol. Genet. Genomics 294, 1343–1357. 10.1007/s00438-019-01584-6 31197471

[B16] Huang Q.-Y. Xu F.-H. Shen H. Deng H.-Y. Liu Y.-J. Liu Y.-Z. (2002). Mutation Patterns at Dinucleotide Microsatellite Loci in Humans. Am. J. Hum. Genet. 70, 625–634. 10.1086/338997 11793300 PMC384942

[B17] HUGO Pan-Asian SNP Consortium, Abdulla M. A. Ahmed I. Assawamakin A. Bhak J. Brahmachari S. K. (2009). Mapping Human Genetic Diversity in Asia. Science 326, 1541–1545. 10.1126/science.1177074 20007900

[B18] Jakobsson M. Rosenberg N. A. (2007). CLUMPP: a Cluster Matching and Permutation Program for Dealing with Label Switching and Multimodality in Analysis of Population Structure. Bioinformatics 23, 1801–1806. 10.1093/bioinformatics/btm233 17485429

[B19] Kong A. Gudbjartsson D. F. Sainz J. Jonsdottir G. M. Gudjonsson S. A. Richardsson B. (2002). A High-Resolution Recombination Map of the Human Genome. Nat. Genet. 31, 241–247. 10.1038/ng917 12053178

[B20] Lang Y. Guo F. Niu Q. (2019). StatsX v2.0: the Interactive Graphical Software for Population Statistics on X-STR. Int. J. Leg. Med. 133, 39–44. 10.1007/s00414-018-1824-6 29564553

[B21] Larue B. L. Lagacé R. Chang C.-W. Holt A. Hennessy L. Ge J. (2014). Characterization of 114 Insertion/deletion (INDEL) Polymorphisms, and Selection for a Global INDEL Panel for Human Identification. Leg. Med. 16, 26–32. 10.1016/j.legalmed.2013.10.006 24296037

[B22] Li L. Ye Y. Song F. Wang Z. Hou Y. (2019). Genetic Structure and Forensic Parameters of 30 InDels for Human Identification Purposes in 10 Tibetan Populations of China. Forensic Sci. Int. Genet. 40, e219–e227. 10.1016/j.fsigen.2019.02.002 30744985

[B23] Lin Z. Kejie W. Hongyan W. Aiying F. Xupeng S. Zhendong Z. (2017). Analysis of Mutation of 20 Autosomal Short Tandem Repeat Loci in Henan Han Population. Chin. J. Forensic Med. 32, 33–35. 10.13618/j.issn.1001-5728.2017.01.009

[B24] Martinez J. Polverari F. S. Silva F. A. D. J. Braganholi D. F. Ferraz J. Gusmão L. (2019). Genetic Characterization of 32 X-InDels in a Population Sample from São Paulo State (Brazil). Int. J. Leg. Med. 133, 1385. 10.1007/s00414-018-01988-w 30612323

[B25] Mills R. E. Luttig C. T. Larkins C. E. Beauchamp A. Tsui C. Pittard W. S. (2006). An Initial Map of Insertion and Deletion (INDEL) Variation in the Human Genome. Genome Res. 16, 1182–1190. 10.1101/gr.4565806 16902084 PMC1557762

[B26] Moriot A. Santos C. Freire-Aradas A. Phillips C. Hall D. (2018). Inferring Biogeographic Ancestry with Compound Markers of Slow and Fast Evolving Polymorphisms. Eur. J. Hum. Genet. 26, 1697–1707. 10.1038/s41431-018-0215-2 29995845 PMC6189140

[B27] Ossa H. Posada Y. Trujillo N. Martínez B. Loiola S. Simão F. (2021). Patterns of Genetic Diversity in Colombia for 38 Indels Used in Human Identification. Forensic Sci. Int. Genet. 53, 102495. 10.1016/j.fsigen.2021.102495 33743518

[B28] Peakall R. Smouse P. E. (2012). GenAlEx 6.5: Genetic Analysis in Excel. Population Genetic Software for Teaching and Research-Aan Update. Bioinformatics 28, 2537–2539. 10.1093/bioinformatics/bts460 22820204 PMC3463245

[B29] Reich D. E. Cargill M. Bolk S. Ireland J. Sabeti P. C. Richter D. J. (2001). Linkage Disequilibrium in the Human Genome. Nature 411, 199–204. 10.1038/35075590 11346797

[B30] Rosenberg N. A. (2004). Distruct: a Program for the Graphical Display of Population Structure. Mol. Ecol. Notes 4, 137. 10.1046/j.1471-8286.2003.00566.x

[B31] Schaffner S. F. (2004). The X Chromosome in Population Genetics. Nat. Rev. Genet. 5, 43–51. 10.1038/nrg1247 14708015

[B32] Sheng X. Bao Y. Zhang J. S. Li M. Li Y. N. Xu Q. N. (2018). Research Progress on InDel Genetic Marker in Forensic Science. Fa Yi Xue Za Zhi 34, 420–427. 10.12116/j.issn.1004-5619.2018.04.016 30465411

[B33] Su B. Xiao J. Underhill P. Deka R. Zhang W. Akey J. (1999). Y-chromosome Evidence for a Northward Migration of Modern Humans into Eastern Asia during the Last Ice Age. Am. J. Hum. Genet. 65, 1718–1724. 10.1086/302680 10577926 PMC1288383

[B34] The 1000 Genomes Project Consortium, Abecasis G. R. Altshuler D. Auton A. Brooks L. D. Durbin R. M. (2010). A Map of Human Genome Variation from Population-Scale Sequencing. Nature 467, 1061–1073. 10.1038/nature09534 20981092 PMC3042601

[B35] Via M. Gignoux C. R. Roth L. A. Fejerman L. Galanter J. Choudhry S. (2011). History Shaped the Geographic Distribution of Genomic Admixture on the Island of Puerto Rico. PloS one 6, e16513. 10.1371/journal.pone.0016513 21304981 PMC3031579

[B36] Wang Y. Lu D. Chung Y.-J. Xu S. (2018). Genetic Structure, Divergence and Admixture of Han Chinese, Japanese and Korean Populations. Hereditas 155, 19. 10.1186/s41065-018-0057-5 29636655 PMC5889524

[B37] Watanabe Y. Naka I. Khor S.-S. Sawai H. Hitomi Y. Tokunaga K. (2019). Analysis of Whole Y-Chromosome Sequences Reveals the Japanese Population History in the Jomon Period. Sci. Rep. 9, 8556. 10.1038/s41598-019-44473-z 31209235 PMC6572846

[B38] Weber J. L. David D. Heil J. Fan Y. Zhao C. Marth G. (2002). Human Diallelic Insertion/deletion Polymorphisms. Am. J. Hum. Genet. 71, 854–862. 10.1086/342727 12205564 PMC378541

[B39] Wen B. Li H. Lu D. Song X. Zhang F. He Y. (2004). Genetic Evidence Supports Demic Diffusion of Han Culture. Nature 431, 302–305. 10.1038/nature02878 15372031

