# Supplementary material for: Genetic Structure and Forensic Feature of 38 X-Chromosome InDels in the Henan Han Chinese Population
Source: Front Genet. 2022 Jan 3;12:805936. doi: 10.3389/fgene.2021.805936 (PMC8762224; doi:10.3389/fgene.2021.805936)
Supplement: Supplementary file 4 [file DataSheet2.DOCX]

**Figure S2**


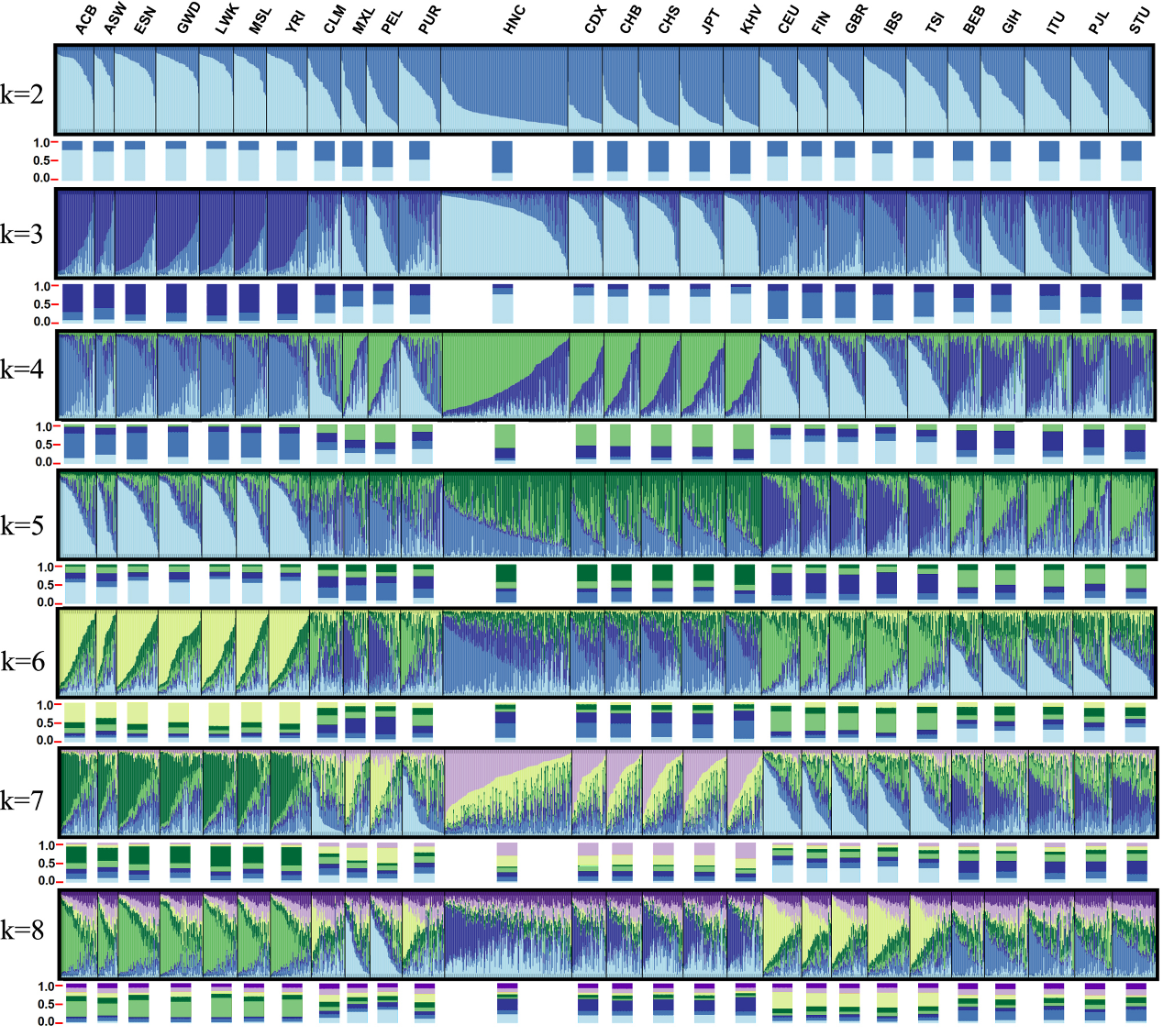
**Structure analysis results for males of the 27 populations:** Each vertical line represents an individual and all individuals were clustered by the populations. Colors represent the inferred ancestry from *K* ancestral populations. Histograms below each population shows the percentage of inferred ancestry in the population.
