# Supplementary material for: Genetic Structure and Forensic Feature of 38 X-Chromosome InDels in the Henan Han Chinese Population
Source: Front Genet. 2022 Jan 3;12:805936. doi: 10.3389/fgene.2021.805936 (PMC8762224; doi:10.3389/fgene.2021.805936)
Supplement: Supplementary file 5 [file DataSheet1.DOCX]

**Figure S1**


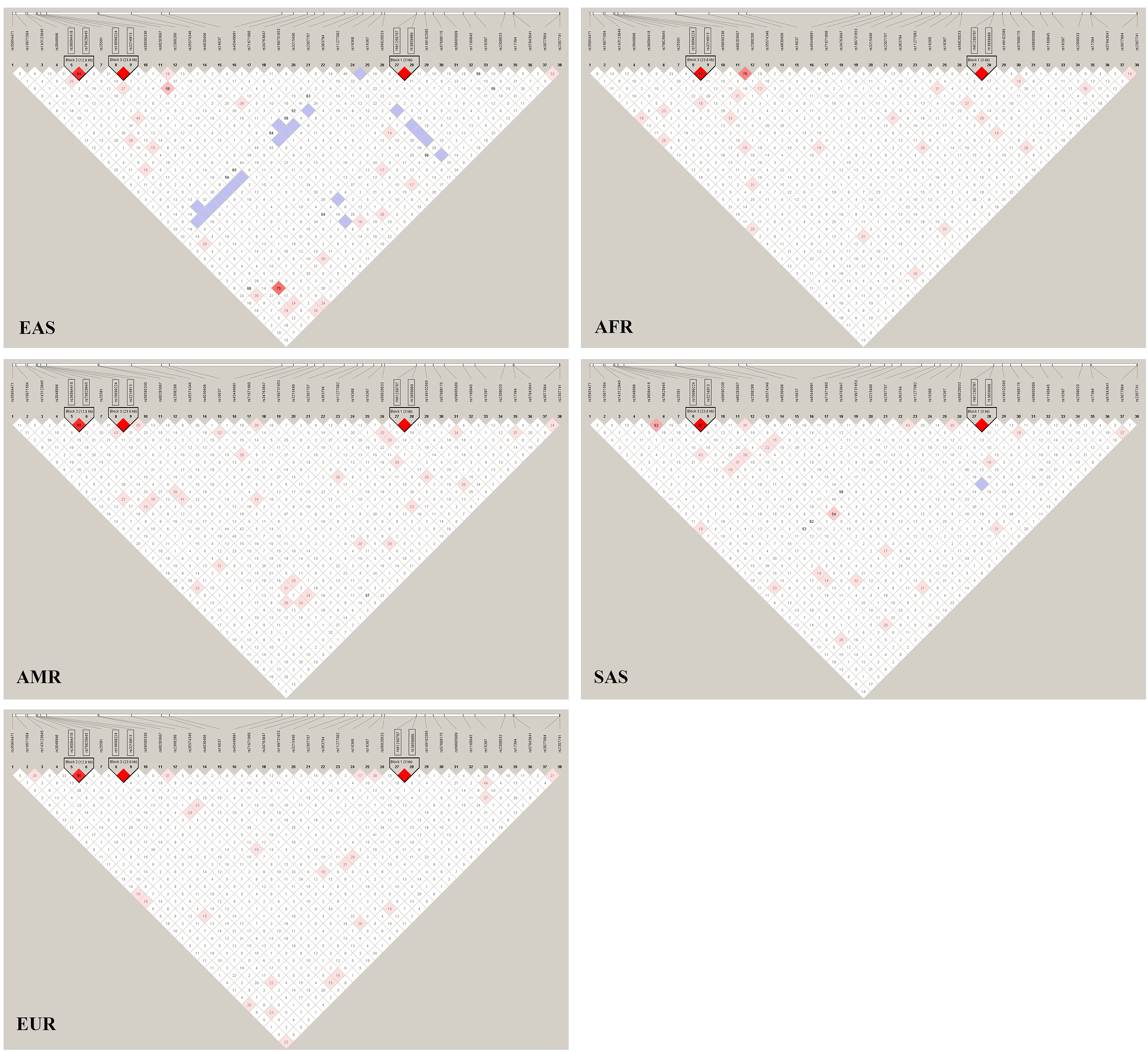
**The degree of LD among the 38 InDel loci in East Asian, African, American, South Asian and European groups. The red color indicates a high level of linkage between two loci.**
